# Single‐Crystal PZT‐Driven Organic Piezo‐Phototronic Adaptive Transistors Toward Advanced Spatiotemporal Visual Computing

**DOI:** 10.1002/advs.202521549

**Published:** 2026-02-03

**Authors:** Chenhao Xu, Xingyu Cao, Zewen Li, Shifu Xiong, Yongxu Hu, Zhongwu Wang, Yujie Yuan, Lei Zheng, Wenping Hu

**Affiliations:** ^1^ Tianjin Key Laboratory of Film Electronic and Communication Devices School of Integrated Circuit Science and Engineering Tianjin University of Technology Tianjin China; ^2^ State Key Laboratory of Crystal Materials Tianjin Key Laboratory of Functional Crystal Materials Institute of Functional Crystals Tianjin University of Technology Tianjin China; ^3^ School of Materials Science and Engineering Tianjin University of Technology Tianjin China; ^4^ Key Laboratory of Organic Integrated Circuits Tianjin Key Laboratory of Molecular Optoelectronic Sciences Department of Chemistry, Institute of Molecular Aggregation Science Ministry of Education Tianjin University Tianjin China

**Keywords:** image sensing, neuromorphic vision technology, organic adaptive memory transistor (OAMT), Organic semiconductor (OPCS), piezo‐phototronic effect

## Abstract

Integrating event detection and grayscale sensing in a single pixel/transistor enables compact, intelligent, flexible neuromorphic spatiotemporal visual imaging. Memory phototransistors based on organic phase‐change semiconductors (OPCSs) are promising due to the high theoretical photo‐sensing‐storage capacity, excellent conductance linearity/symmetry, and intrinsic flexibility. However, such systems are constrained by low phase‐change efficiency (narrow memory window/capacity) arising from weak and poorly controllable organic molecular interactions, restricting complex feature extraction and increasing energy consumption during information perception and processing. Here, we propose a single‐crystal PZT‐driven piezo‐phototronic organic adaptive memory transistor (OAMT) with optimized stress distribution and multi‐field control, significantly enhancing molecular conformation transition efficiency under low‐power operation. The device achieves a record memory window capacity factor (*γ*) of ∼0.87 at a subthreshold swing (*SS*) of 200 mV/decade, with over 90% recognition accuracy from the OAMT device's actual LTP/LTD synaptic functions in neuromorphic simulations. Furthermore, the device's adaptive multistage phase‐transition behavior in response to varying UV pulse densities enables stable current changes—transitioning from molecular conformation 2 to mixed conformations (1+2) in the PCS layer—as well as transient current spikes from conformation 2 to 1. The device simulates real‐time flight attitude and dynamic grayscale detection via precise spatio‐temporal synchronization, showing great potential for advanced visual technology.

## Graphical abstract

Here, we propose a single‐crystal PZT‐based piezo‐phototronic organic adaptive memory transistor (OAMT), achieving a record memory window capacity factor (γ) of 0.87 at a low SS of 200 mV/decade via efficient multi‐field control. The device achieves a high recognition accuracy ∼ 90% in neuromorphic simulations, demonstrates robust fault tolerance under over 30% noise, and enables simultaneous capture of dynamic events and grayscale information.

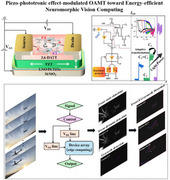

## 1 Introduction

Advancements in the neuromorphic spatiotemporal visual system (NSVS) are crucial for improving the performance and reliability of robots and autonomous vehicles in real‐world environments [1]. Dynamic and active pixel vision sensors (DAVIS) are notable for its ability to integrate both event detection, as found in traditional active pixel sensors, and grayscale detection, characteristic of event‐driven dynamic vision sensors, within a single pixel. [2] Event detection captures dynamic motion and static background information by recording all pixels at a fixed rate; moreover, grayscale detection identifies changes in light intensity with high temporal and spatial resolution. However, the application of DAVIS in highly integrated and flexible NSVS remains challenging due to its complex multi‐component pixel structure and high power consumption. Phase‐change memories (PCMs) offer a present opportunity in aforementioned emerging fields due to their large memory window/capacity, excellent photoelectric tunability, high linearity and symmetry in conductance modulation, and low power consumption. [3, 4, 5] In contrast to traditional inorganic semiconductor layers, such as Ge_2_Sb_2_Te_5_ and Sc_0.2_Sb_2_Te_3_ [6, 7, 8], PCMs based on organic phase‐change semiconductors (OPCSs) show greater potential for next‐generation flexible memory technology and NSVS [9]. This is due not only to their intrinsic flexibility, which provides unique advantages, but also to their high degrees of freedom for twisting and tuning via multi‐field modulation (e.g., light, electric, thermal). This results in higher phase‐change efficiency and a larger memory window/capacity, enabling efficient information perception and processing under low power consumption [9, 10]. However, a major challenge lies in the inherently weak intermolecular interactions among organic molecules, resulting in a lack of effective strategies to achieve reversible phase transitions with high efficiency [11].

The piezo‐phototronic effect has been revealed to be an effective and reliable strategy for modulating optoelectronic processes through the tuning of energy band structures and carrier transport behavior. Generally, the introduction of appropriate stress into semiconductors can modulate their physical properties and enhance the optoelectronic performance of their‐based electronic components, has been demonstrated in the design and fabrication of energy‐efficiency devices such as solar cells, light‐emitting diodes, and photodetectors [12, 13, 14, 15, 16]. For example, recent studies have demonstrated the mechanical tunability of the photoelectric properties in semiconducting MoS_2,_ which have been realized by the uniform stress generated by a piezoelectric substrate [17] or nonuniform stress induced by bending the substrate or exploiting the ultrasharp indenter [18, 19, 20], respectively. In addition, based on the polycrystalline PZT‐based (lead zirconate titanate) piezoelectric substrate, a nanoelectromechanical field‐effect transistor (NEM‐FET) with an ultralow subthreshold swing (*SS*) as a contact‐based switch is also demonstrated [21]. The voltage‐induced nonuniform stress can act in the semiconductor and further control the contact between the electrode and the semiconductor. The *SS* characterizes the steepness of the device's switching behavior in the subthreshold region: a smaller slope indicates a smaller change in gate‐source voltage is required to transition between the off and on states, resulting in faster switching speed and lower power consumption. These studies provide valuable guidance for designing intelligent OPCS‐based memory transistors with stress‐assisted phase‐change mechanisms for low‐power neuromorphic visual electronics.

Herein, a novel piezo‐phototronic modulated organic adaptive memory transistor (OAMT) based on a single‐crystal PZT piezoelectric medium is demonstrated, utilizing OPCS of 3,6‐Bis(anthracen‐2‐yl)thieno[3,2‐b]thiophene (3,6‐DATT) with reversible molecular conformation transitions. Due to the efficient phase‐change responses of 3,6‐DATT to stress, electric fields, and UV light, the devices achieve a record memory window capacity factor (*γ*) of ∼0.87 and a high current ON/OFF ratio exceeding 10^5^ under a subthreshold swing (*SS*) of 200 mV/decade. Moreover, they support fast programming/erasing speeds (∼2 s) and long retention times beyond 10^4^ s. This achievement outperforms most of the reported organic memristors. Additionally, it achieves synaptic plasticity like potentiation/depression with highly linear symmetric conductance (∼ a fitted *R* ^2^ of 0.99) and enables a high recognition accuracy of ∼ 90% with strong fault tolerance over 30% noise ratio. Impressively, the device leverages its adaptive phase‐transition behavior to simultaneously detect stable current changes and transient current spikes. As a result, the device enables in‐memory photo‐sensing and computing to extract static background information through convolutional processing with programmable kernels, while also supporting synchronous detection of dynamic and static images and grayscale data within a single transistor. The results demonstrate that the OAMT devices hold significant potential as a promising paradigm for future NSVS.

## 2 Results and Discussion

### 2.1 Device Visualization

**FIGURE 1:**
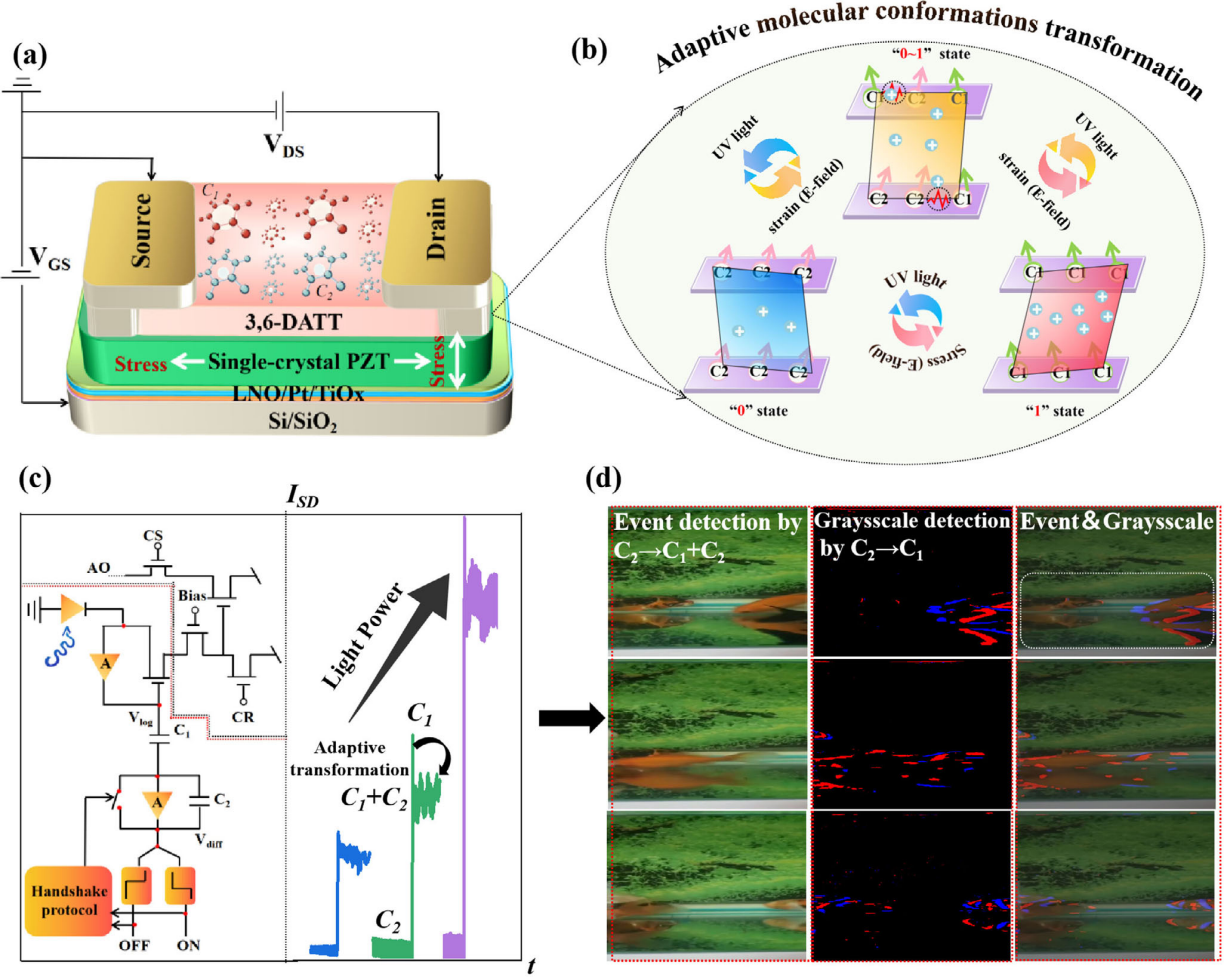
Piezo‐phototronic effect‐modulated OAMT enabling synchronous detection of events and grayscale information. (a) Design of the OAMT. (b) Molecular conformations transformation characteristics of 3,6‐DATT by stress, UV light and electric field (*E*). (c) Pixel circuit of traditional DAVIS and the adaptive optoelectronic behavior of the OAMT device. (d) Synchronous detection of both dynamic and static images enabled by the developed devices.

**FIGURE 2:**
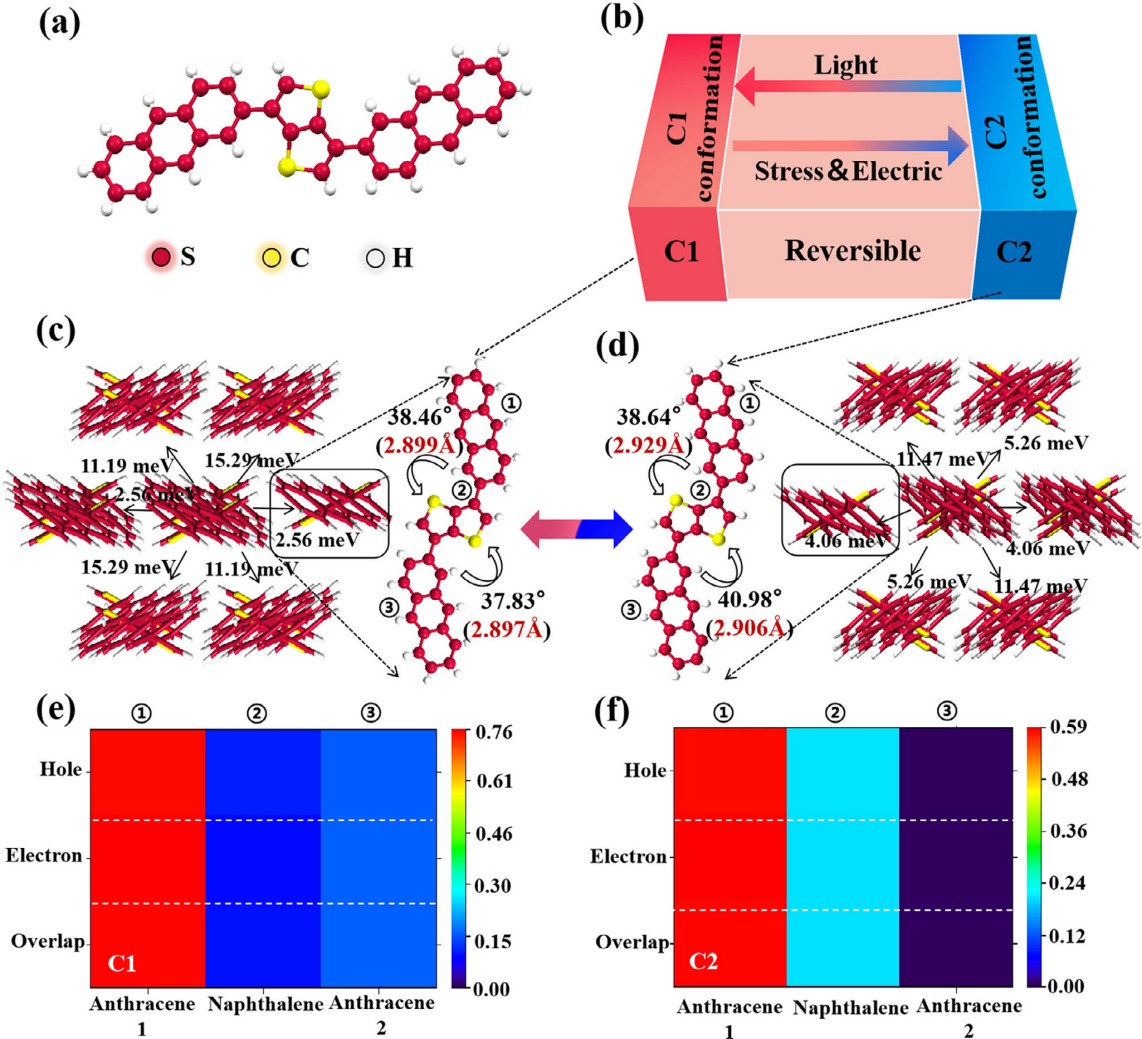
Different crystalline structures of 3,6‐DATT formed by two molecular conformations. (a) The molecular structure of 3,6‐DATT. (b) Reversible transformation of two crystalline phase under external stimulis. (c, d) The crystal structures and molecular conformations of two crystalline phase (CCDC numbers: 2121604 and 2125256). (e, f) The mappings of each molecule (group) contributing to electrons and holes of C1 phase and C2 phase, which the 1, 2, 3 are corresponding to the anthracene group, thieno[3,2‐b]thiophene group and another anthracene group.

Figure 1a schematically explains the operation of our designed piezo‐phototronic effect‐modulated OAMT, which enables a single transistor structure to integrate both event detection and event‐driven grayscale sensing simultaneously. The core design concepts of our developed OAMTs are mainly the following two aspects: i) The OPCS of 3,6‐DATT serves as the channel material, featuring torsion angles between the two aromatic planes that are well reversibly modulated via external stimulus (e.g., light, electric, stress, Figure 1b). The molecular conformational transition induces crystalline polymorphisms, including the C1 and C2 phases, which exhibit significantly different charge transport properties [10]. The details of how these structures influence charge transport will be discussed in detail later (Figure 2). The subtle nature of this conformational change ensures the reversibility of the phase transition and associated electrical properties, thereby enabling high‐performance organic phase‐change memory devices. ii) A single‐crystal PZT piezoelectric film is employed as the gate dielectric, which enables optimized stress distribution resulting from the inverse piezoelectric effect—a phenomenon in which the application of an electric field (or voltage) to the surface of a piezoelectric material induces mechanical deformation, such as elongation or contraction, along the direction of the field [21]. To obtain a single‐crystal gate dielectric of PZT film with 1.5 µm that functions as the source of piezoelectrically driven uniform stress, a lanthanum nickelate (LNO) buffer layer of 25 nm is pre‐prepared on the Pt‐based substrate. Such a system significantly enhances the molecular conformation transition efficiency of OPCS under low‐power operation. Details about fabrication and characterization processes of these devices can be found in the experimental section. As shown in Figure 1c, the DAVIS circuit schematic typically requires dozens of transistors, which constrains integration density and increases power consumption. In contrast, the single 3,6‐DATT OAMT channel transitions from C2 to C1 under UV illumination and shows a differential response to varying light power densities, producing transient current spikes for dynamic grayscale tracking. Meanwhile, the PZT‐enabled stress‐assisted biasing effect induces a transition to a C1+C2 state, producing stable current changes for the event's information sensing (Figure 1d). This would guarantee seamless integration of data and eliminate any temporal inconsistencies.

### 2.2 Material and Structural Characteristics

The *p*‐type 3,6‐DATT structures of two crystalline phases (CCDC numbers: 2121604 and 2125256), consisting of two different molecular conformations, exhibit nearly identical packing arrangements, with only a minor difference in the torsion angle, yet display significantly different charge transport properties (Figure 2a,b). As shown in Figure 2c,d, the torsion angle between anthracene and thieno[3,2‐b]thiophene increases from 37.83° in the C1 conformation to 40.98° in the C2 conformation, while the S···H bond distance changes from 2.897 Å in C1 to 2.906 Å in C2, leading to a change in spatial configuration. The torsion angle of the other side increases from 38.46°C to 38.64°C with the S‐H distance from 2.899 Å to 2.929Å. Then, by calculating their reorganization energy and transfer integrals, we find that the C1 phase had a larger transfer integral than the C2 phase, indicating superior orbital overlap and charge transport performance of C1. The theoretical *p*‐type mobilities of two phases can further be calculated by MOMAP, which the value of C1 is two times than C2. To obtain an in‐depth understanding of the difference in charge‐transport properties, more comprehensive theoretical calculations are carried out for the two phases of 3,6‐DATT. All electronic configuration analyses are obtained by DFT and time‐dependent DFT (TD‐DFT) calculations, in which the ωB97XD functional is used at the def 2‐TZVP basis set and using the restricted DFT method for S0 state, TD‐DFT for S1 state [22]. All calculations are carried out using the Gaussian 16 package, and excited‐state performance analysis is conducted with Multiwfn [23]. In addition, we use the CVFF force field of the Forcite module in Materials Studio to directly calculate the lattice energy of organic crystal structures [24]. For the lattice energy, the C1 is lower than that of the C2 by 0.62 eV, indicating the possibility of conformation conversion between C1 and C2 under additional energy supplements/escape. Then, we select the molecular chains with a typical torsion angle in the organic crystal structure for electron‐hole correlation analysis. Holes are enriched in the blue region and electrons in the green regions of C1 and C2, as shown in the electron‐hole distribution isosurface with value = 0.001 (Figure S1a). To more clearly display the distribution of carrier of two conformations, are used to describe the distribution of holes and electrons using a Gaussian, effectively smoothing out distribution details (Figure S1b). The equivalent map of holes and electrons becomes elliptical, which distributes more along the long axis than along the other directions in C1 and C2. The heatmaps of each molecule contributing to electrons/ holes of the C1 and C2 are shown in Figure 2e,f. From the heatmap images, C1 shows a smaller overlap degree than C2. This indicates a greater separation distance between holes and electrons, with holes exhibiting higher mobility than electrons. Moreover, the calculated hole and electron delocalization index [25] (HDI and EDI) of C1 and C2 are 1.15 and 1.20, indicating a higher hole precipitation and hole carries transfer efficiency in C1. The mathematical formulas used in the qualitative analysis of *p*‐type 3,6‐DATT are listed in the experimental section. These results lay the theoretical basis of designing and synthesizing novel OPCS materials.

**FIGURE 3:**
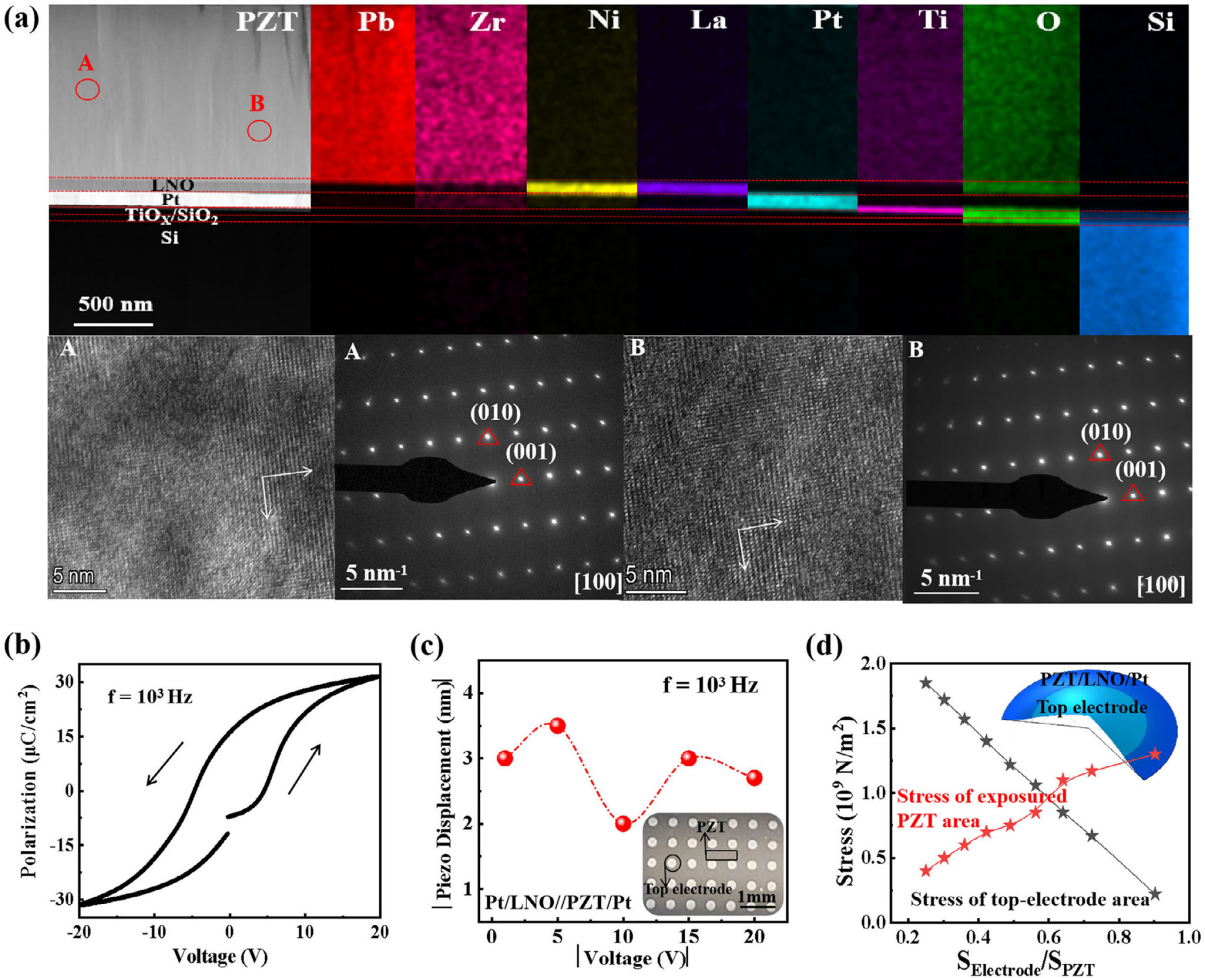
Structural characterizations of piezoelectric stress module. (a) Cross‐sectional image and EDS mapping of a single‐crystal PZT enabled piezoelectric stress module, which mainly includes 25 nm TiO_2_, 100 nm Pt, 100 nm LNO, 1.5 µm PZT and 25 nm 3,6‐DATT film, respectively. In addition, it shows the HRTEM and SAED patterns of the single‐crystal PZT film. (b) Typical *P–E* curve measured at 10^3 ^Hz of a 300 × 300 µm^2^ PZT film. (c) Displacement as a function of the electric field for the 1 um PZT film at a frequency of 1000 Hz. (d) The FEM simulation results of stress distribution within a Pt‐PZT‐LNO/Pt device (insert) under different area ratios between the top electrode and PZT.

**FIGURE 4:**
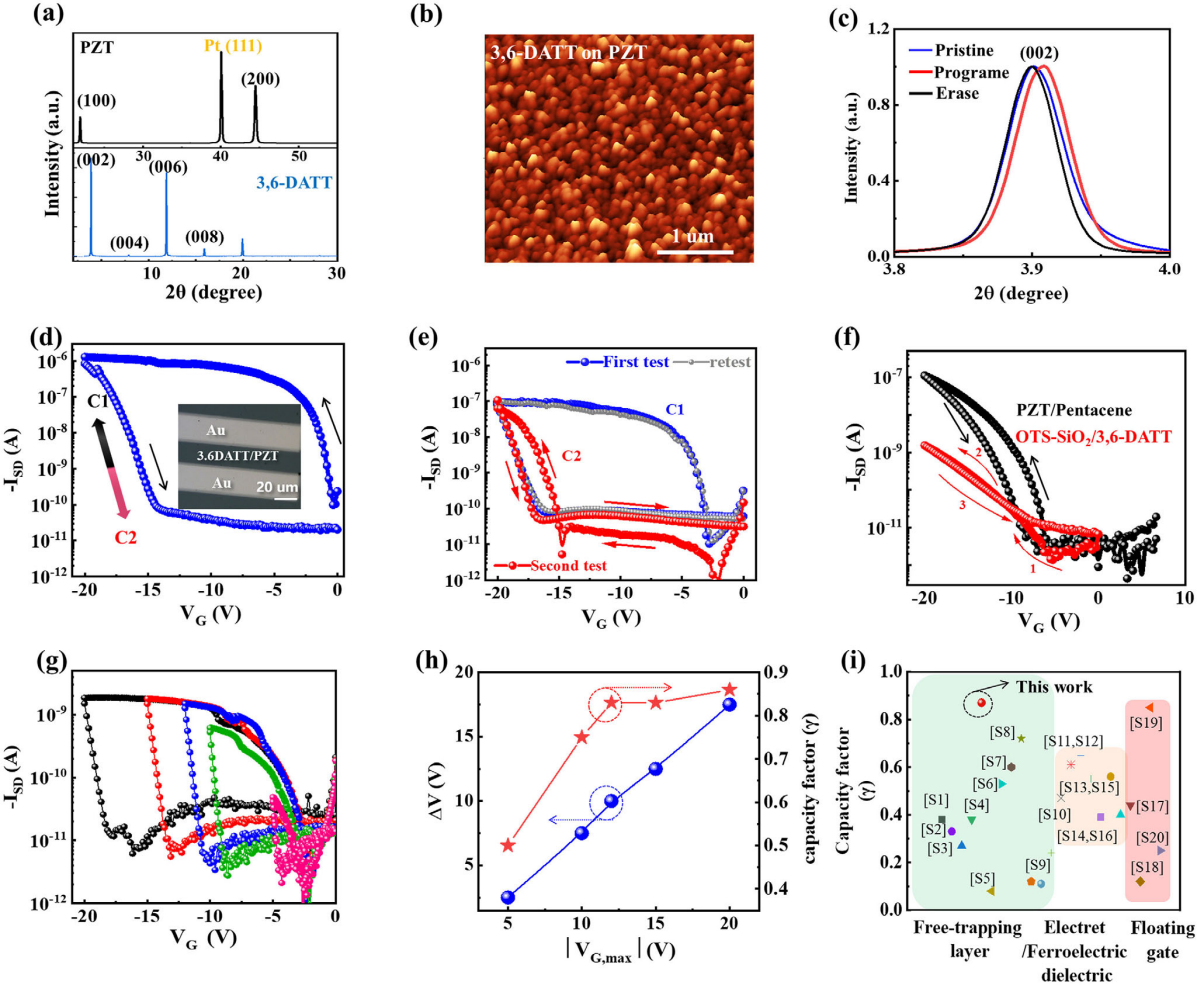
Memory characteristics of the piezo‐phototronic OAMT. (a) Typical X‐ray diffraction of the single‐crystal PZT and 3,6‐DATT thin film. (b) Three‐dimensional AFM image of the 3,6‐DATT thin film on the PZT‐based substrate. (c) In‐plane XRD patterns of 3,6‐DATT film before and after external stimuli. d) Typical transfer curves at a *V_SD_* = −20 V and optical image of the devices with a large hysteresis window. (e) The difference in transfer curves of the piezo‐phototronic modulated memory measured at sequential two tests, revealing the changed channel from C1 phase (black line) to C2 phase (red line). The blue line is the reset operation by the UV illumination which the device channel changed from C2 to C1. (f) Transfer curves of the 3,6‐DATT‐based OFETs on the OTS‐modified SiO_2_/Si substrate and Pentacene‐based OFETs on the PZT substrate, showing negligible hysteresis effect. (g) Hysteresis loops measured at different *V_G_* conditions and resetting by an UV illumination after each test. (h) Extracted memory window (left axis) and calculated capacity factor (right axis) as a function of *V_G, max_*. (i) Comparison of the memory characteristics of representative OMT based on different device structure.

The cross‐sectional image of the PZT‐based substrate and distribution of Pb, Zr, Ni, La, Pt, Ti, O, and Si using energy‐dispersive spectroscopy (EDS) mapping, showing a specific distribution of each layer and excellent inter‐layer contact (Figure 3a). The single‐crystal properties of the PZT layer are characterized using high‐resolution transmission electron microscopy (HRTEM) and selected‐area electron diffraction (SAED). It takes along the [100] direction and reveals a consistent morphology across the entire selected region (from A to B) and confirms the PZT's single‐crystalline properties. The pure perovskite phase with (h00) texturation is recognized for exhibiting the highest transverse piezoelectric coefficient at the morphotropic phase boundary in the (100) crystallographic orientation, thereby facilitating the realization of an efficient and uniform stress source [26, 27]. Moreover, we characterize the ferroelectric and dielectric properties of the PZT single‐crystal film using a sandwich structure of Pt‐PZT/LNO‐Pt with a 300 µm effective diameter. The *P–E* curve measured at 1000 Hz exhibits a typical hysteresis shape, characteristic of ferroelectric materials (Figure 3b). The polarization value reaches 30 µC/cm^2^ under a voltage of 20 V (Figure 3c), with a remnant polarization (2*P_r_*) of approximately 30 µC/cm^2^ and a coercive field (2*E_c_*) of around 66 kV/cm. The capacitance‐frequency (*C*–*F*) curves of the 1.5 um PZT film measured at 1000 Hz∼10000 Hz are shown in Figure S2, where the capacitance is stable around 0.4 µF cm^−2^ ranged a broad frequencies. In addition, a large‐signal displacement response of the single‐crystal PZT is measured on a clamped 9 × 10^−4^ µm^2^ capacitor under 1000 Hz, showing the maximum effective displacement up to 3.5 nm at a 5 V bias. To further understand the PZT‐enabled stress distribution on organic semiconductors, we use the finite element method (FEM) simulation (Figure 3d) of stress distribution by changing the area (*S*) ratios between the top electrode and PZT film. The simulation result shows an increasing stress strength on the exposed PZT area by increasing the ratios of *S*_Electrode_/*S*_PZT_, providing guidance for designing the piezo‐phototronic modulated device. The out‐of‐plane XRD scanning range for the single‐crystal PZT layer is from 10 ° to 60°, covering all possible orientations of the PZT crystal. A significant PZTdiffraction peak of (100) is observed at 21° (Figure 4a, up), and no other crystal orientations are detected at other angles, further confirming the ultrahigh crystallographic quality of the films. Subsequently, the prepared 3,6‐DATT thin film (Figure 4a, below) reveals a series of very sharp and strong diffraction peaks, revealing the ultrahigh crystallographic quality. The AFM image of 3,6‐DATT polycrystalline film shows an island‐shaped stacking structure on the PZT film (shown in Figure 4b). Furthermore, in situ XRD testing confirms that the dynamic transition from C1 to C2 is driven by PZT‐induced stress, and the reverse process by UV irradiation (Figure 4c and Figure S3). The results provide a solid material foundation for the design and construction of high‐performance OAMT devices.

### 2.3 Photoelectric Performance of Piezo‐phototronic OAMT and Charge‐transport Mechanism

Figure 4d,e illustrate the saturated transfer curve of the fabricated piezo‐phototronic modulated OAMT, which exhibits a large hysteresis memory window and shows the device's two core functions: electric field programming (stress/voltage‐induced phase transition) and UV photo‐erasure (light‐induced phase retransition). To effectively demonstrate the optimal performance of each function, we selected two representative devices for illustration. The device features a channel length/width of 20 µm/80 µm, with an optimized stress distribution across the device channel derived from FEM simulation results. In general, hysteresis in the transfer curve is closely related to the underlying mechanism of memory devices, which the large anticlockwise hysteresis window usually means abundant carriers can be captured and released for the traditional charge trap memory [28]. Unlike the aforementioned memories, the large hysteresis behavior of our developed piezo‐phototronic modulated device is attributed to the PZT‐enabled phase‐change behaviors of 3,6‐DATT, where the high resistance state (HRS) of C2 channel is the “off‐state” and the low resistance state (LRS) of C1 channel refers to “on‐state”. Controlled experiments are conducted to clarify the unique hysteresis behaviors of these devices. Firstly, a marked difference is subsequently observed in the *I*–*V* characteristics of PZT‐based device during sequential testing (as illustrated in Figure 4e). This difference further highlights the hysteresis phenomenon inherent to these PZT‐based devices, which arises from a stress‐assisted phase‐change mechanism transitioning from C1 to C2. Moreover, the channel can also be reset by the UV illumination, with the channel changing from C2 to C1, displaying the nature of memory behavior. Moreover, two types of FETs are fabricated, which involve the 3,6‐DATT‐based transistors onto the bare SiO_2_‐Si substrate and the pentacene‐based device on the PZT‐based substrate. These devices don't show an obvious hysteresis effect similar to the developed piezo‐phototronic modulated memory device (Figure 4f). The results demonstrate that the memory property of such OAMT is not derived from the traditional working mechanism, such as the charge trapping effect occurring in the interface between the PZT and semiconductor layer or the ferroelectric switching behavior generally with a clockwise memory window. [29] On the other hand, compared with the Si‐based device with a larger applied *V_G_* bias, the OMAT device shows a significantly lower subthreshold slope (*SS*) of less than 200 mV/dec compared to other transistors with the same Ion/Ioff ratio (10^4^) and operating voltage (−20 V), outperforming similar devices (Table S1). The results show that OMAT device achieves faster turn‐on speed characteristics at lower operating voltages. This is essential for achieving low power consumption during the switching process. These findings underscore the device's considerable potential for applications in low‐power neuromorphic systems.

The hysteresis loops tested by different *V_G_* and *V_SD_* conditions are drawn in Figure 4g and Figure S4, which the device needs to be reset after each test by an UV illumination. The distinct transfer characteristics and tunable memory window highlight the reproducibility and adjustability of these piezo‐phototronic memory devices. Memory window with calculated capacity factor (*γ*), as the most important parameter to distinguish the information storage level [10], is extracted from transfer curves as a function of *V_G, max_* (Figure 4h). In general, the memory window refers to the threshold voltage shift (*∆V_T_*) between the program and erase states or the hysteresis‐induced voltage window (*∆V*), enabling efficient data storage and processing. It can be evaluated using the *γ*, defined as the ratio of the memory window to working voltage. It is noteworthy that our developed memories show the *∆V* and *γ* increase linearly for widening *V_G_* ranges*_,_* and the *γ* value reaches a record 0.87 at a 20 V sweeping rang and low subthreshold swing (*SS*) of 200 mV/decade. These remarkable memory characteristics are significantly superior to those of current organic field‐effect transistor (OFET) memories, as illustrated in Figure 4i and Table S1, particularly for devices utilizing free‐trapping layers where the bare organic semiconductors serve as the charge storage medium, showing a great potential for achieving highly integrated memory circuits with a simple device construction.

**FIGURE 5:**
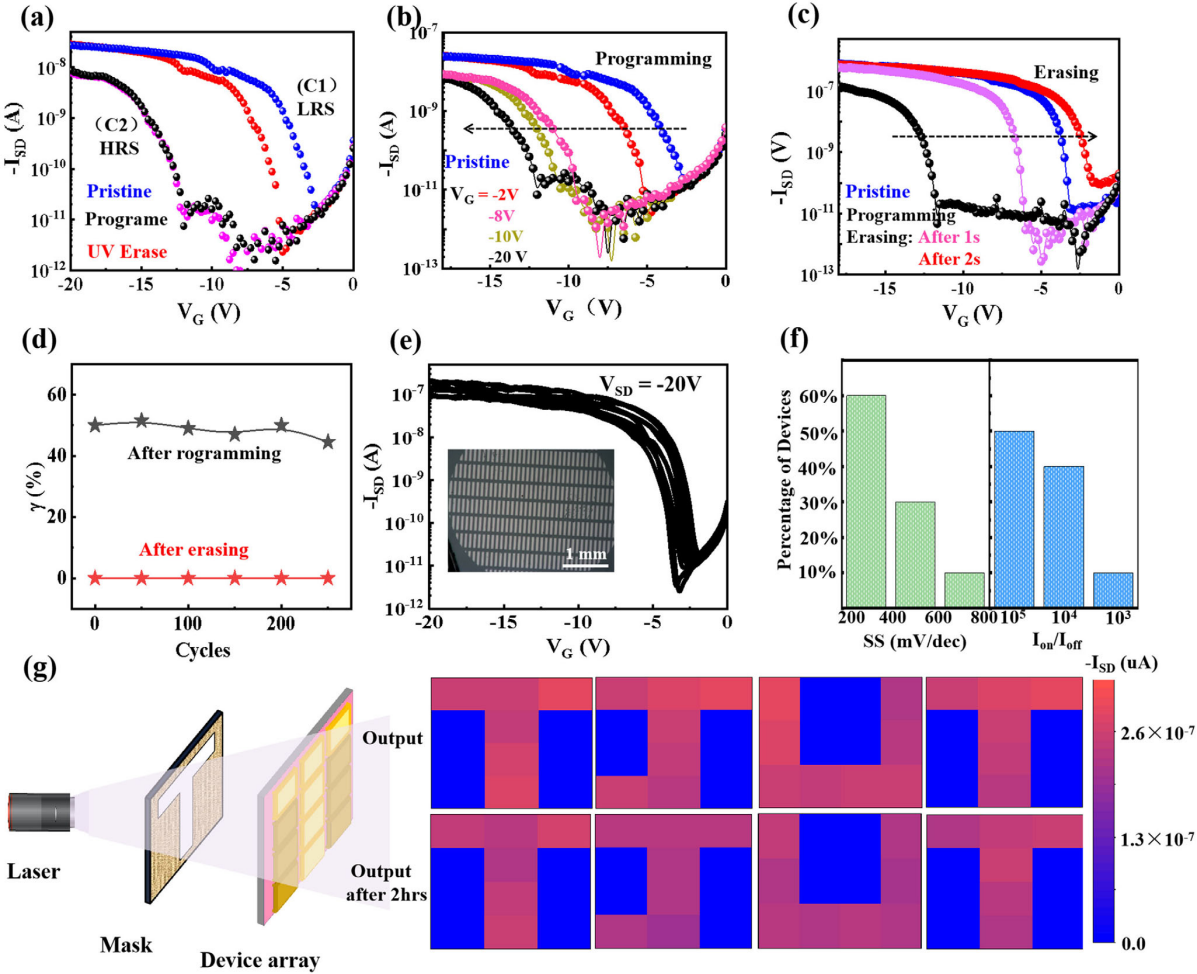
Photoelectric modulation of the OAMT array. (a) Transfer curves of the device at a *V_SD_* = −20 V, including pristine state (blue line), after programming (black line: *V_G_* = −20 V for 2 s), retention time test (pink line: over 10^4^ s reading at *V_G_* = −20 V) and erasing test (red line: UV illumination for 2s under a 21.8 P light power (1 p = 1 µW cm^−2^)). (b) The dependence of *V_T_* shift on the different programming conditions (c) The dependence of *V_T_* shift on the different erasing conditions. d) The cyclability of the piezo‐phototronic modulated OAMT. (e) Large‐area piezo‐phototronic modulated OAMT array devices. (f) The *SS* and *I_on_/I_off_* distributions based on 40 devices, respectively. (g) Image detection of the 3 × 4 arrays with increasing output time and different input images of letter T, J, U T. All the tests were carried out under UV light stimulation (erasing operation). The blue shading regions represent the channel current after programming operations of *V_G_* =−20 V for 2 s and the pink shading regions represent the channel current after erasing operation of UV erasing for 2 s.

To reveal the dynamic phase‐change behaviors and in‐depth charge‐transport mechanism of the device, we draw the schematic diagram of the device’ work mechanism and show the varies transfer curves for the phase‐change process. These curves are tested in the following sequence (Figure 5a–c): (1) pristine state (in the dark environment, blue line), (2) programming (*V_G_* = −20 V for 2 s under dark, black line), and retention test of the devices after programming during almost 3 hrs (pink line). (3) erasing by a 365 nm UV illumination with light density of 21.8 µW cm^−2^ for 2s (red line). After 2s programming operation, the channel current of the device significantly decreases with a large negative shift of threshold voltage (*V_T_*), indicating the switched channel from C1 to C2. Afterward, the curve retraces back to the pristine state (from C2 to C1) only by 2 s erasing illumination, which shows that the conformational transition exhibits a fast response and recovery rate, resulting in the memory device's typical switching behavior. These memory functions of 3,6‐DATT‐based OAMT are superior to the devices fabricated on the Si substrate [11]. Moreover, we carry out continuous multiple measurements of transfer curves to evaluate the stability of the OAMT device. The negligible shift in the programming curves displays a high retention time over 10^4^ s at a constant drain‐source bias (*V_SD_*) of −20 V. In Figure 5b,c, the programming/erasing process and memory window could be also be well adjusted with appropriate gate bias or illumination time, showing the flexible dynamic memory processes and multilevel storage characteristics. Subsequently, we conducted an endurance test and observed reproducible switching over 250 cycles (Figure 5d). Extrapolation based on a model fitted to these 250 cycles indicates that γ above 20% can be sustained beyond 1,600 cycles (Figure S5), demonstrating good cyclability. Forty piezo‐phototronic modulated OAMTs are randomly selected from the array, and their measured transfer curves are shown in Figure 5e. All the devices exhibit similar electrical characteristics with a small device‐to‐device variation. Moreover, the value of *SS* and high on/off ratio (*I_on_/I_off_*) distributions of these devices are collected as shown in Figure 5f, where over 60% of the devices exhibit an SS below 400 mV/dec and over 50% show a high I_on_/Ioff ratio exceeding 10^5^. With integrated photoelectric conversion and retention capabilities, the prepared memory devices show potential for image sensing and memorization. A 3 × 4‐pixel array device is used to detect and memorize the input letter image of T, J, U, T, which the devices were first initialized by *V_G_* = −20 V for 2 s and then written by UV illumination for 2 s (Figure 5g). The letters optical projection patterns are successfully perceived and stored in the array as pixel points, with confirmed by the current distribution read from each pixel, which corresponds to two conductance states (C1 and C2). The current of devices increases under the illumination conditions, and the letters were perceived by the array device. After turning off the light, an obvious current difference between irradiated and non‐irradiated devices can be maintained, suggesting in situ memorization. The input images of continuous operation for 1 h are shown in Figure S6. In our work, the memory device demonstrates stable continuous operation for at least 2 h with only a slight decrease in current, indicating that the PZT‐enabled OAMT possesses robust image perception and storage capabilities. These interesting results could make us deeply understand the underlying conformation‐property relationship in phase‐change organic semiconductors and provide guidance for designing low‐power advanced transistor memory with high‐integration and multifunctionality.

### 2.4 Devices Arrays for Synchronous Dynamic and Static Image Detection

**FIGURE 6:**
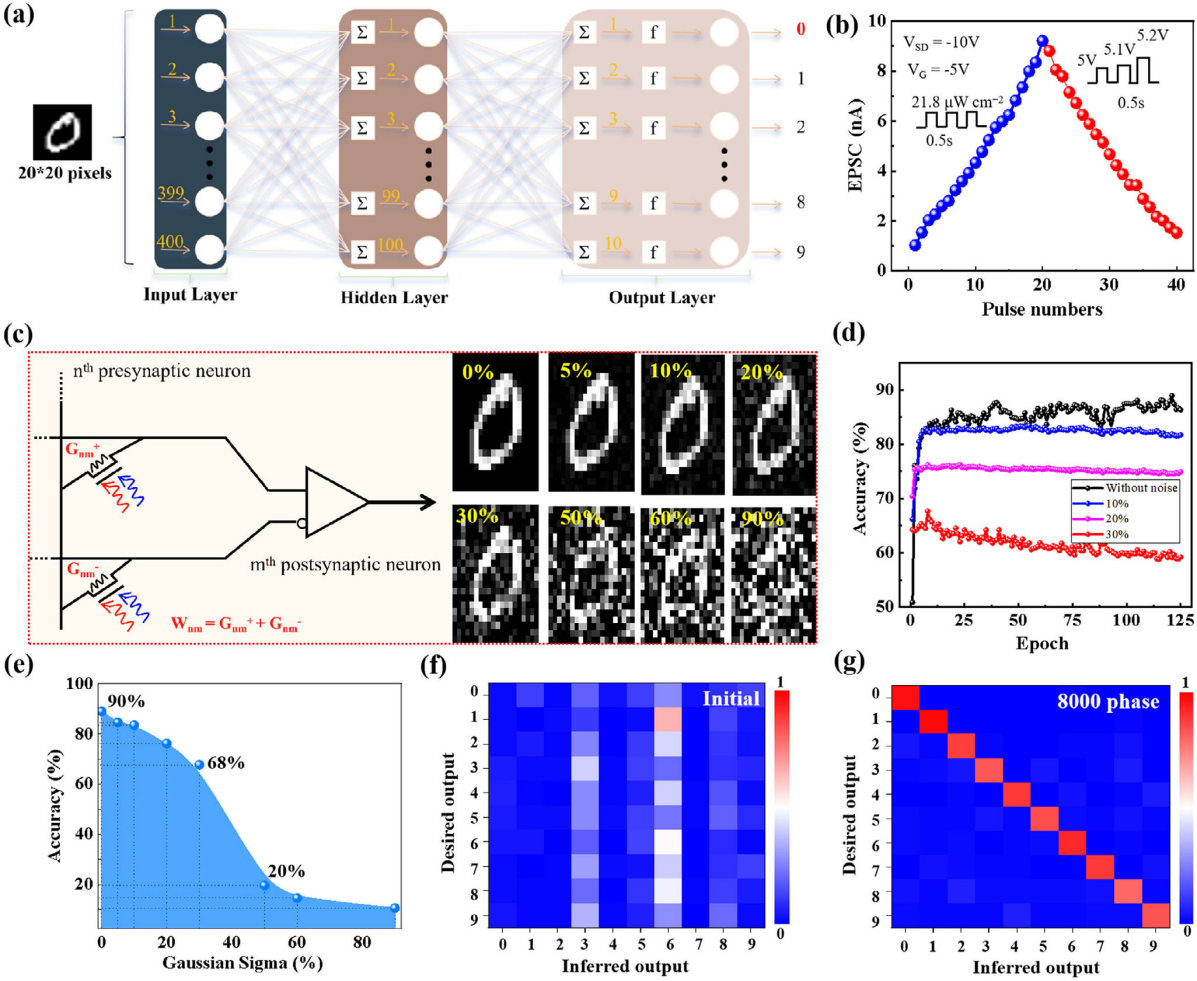
The capacity of neuromorphic computing for piezo‐phototronic OAMT. (a) Schematic of the constructed ANN for handwritten digit “0” recognition process. b) LTP/LTD behavior. (c) Schematic of the synaptic weight defined as the conductance difference of two equivalent optical synapses, which the handwritten digits are with different noise ratios (0%–90%). Recognition accuracy of the network in the flat state as a function of (d) training epochs and (e) noise pixel proportion. (f, g) Confusion matrices displaying recognition results before training and after training.

To study the neuromorphic computing capability of the piezo‐phototronic modulated OAMT, an ANN is simulated based on stable current/conductance levels shown in Figure 6a, and a Modified National Institute of Standards and Technology (MNIST) visual object recognition system is performed [30]. The flexible memory characteristics validate the existence of multiple intermediate states, which is the core foundation for devices to achieve neuromorphic computing. The device undergoes a slow state transition when light and electrical stimuli are modulated, exhibiting artificial synaptic characteristics during this process. To represent the number of simulated input neurons, 20 × 20 pixels are normalized and converted into a 400 × 1 one‐dimensional array. The network includes 100 hidden neurons connected to the input and output layers, with 10 output neurons corresponding to the 10 digit classes in the MNIST dataset. All neurons are mapped to the synaptic array nodes of OAMTs. As shown in Figure 6b and Figure S7, long‐term potentiation/depression (LTP/LTD) of synaptic weights is induced by 20 consecutive light pulses (21.8 µW cm^−2^, 0.5 s, no electric pulses) and and 20 increasing electric pulses (no light pulses), achieving high linearity with a fitted *R* ^2^ of 0.99 and excellent gain/attenuation symmetry in conductance modulation. The performance of LTP and LTD provides key parameters for neuromorphic simulation. The maximum and minimum conductance values in the LTP and LTD processes represent the modulation range of weights in neuromorphic computing. The nonlinearity, deriving from the nonlinear fitting of conductance variation during the LTP/LTD, and the number of training pulses jointly determine the number of distinguishable states and the degree of discrimination in neuromorphic computational weights. Moreover, different levels of noise (0%–90%) were applied to the database (Figure 6c) to show the fault‐tolerance capability of the network. The overall identification accuracy of both devices initially increased rapidly and then gradually saturated during training, reaching approximately 90% (Figure 6d), signifying a notable application prospect in neuromorphic computing. Figure 6e illustrated the recognition accuracies of the network for handwritten digits across various noise ratios. Notably, the recognition accuracy remained above 60% even when the noise ratio was set to 30%, highlighting the network's robust fault‐tolerance capability. Figure 6f,g depicted a visualized learning process in the ANN system, wherein the maximum inferred output value within each row progressively aligns with the expected output value within each column. This shows that our devices successfully identified handwritten digits from the MNIST dataset.

**FIGURE 7:**
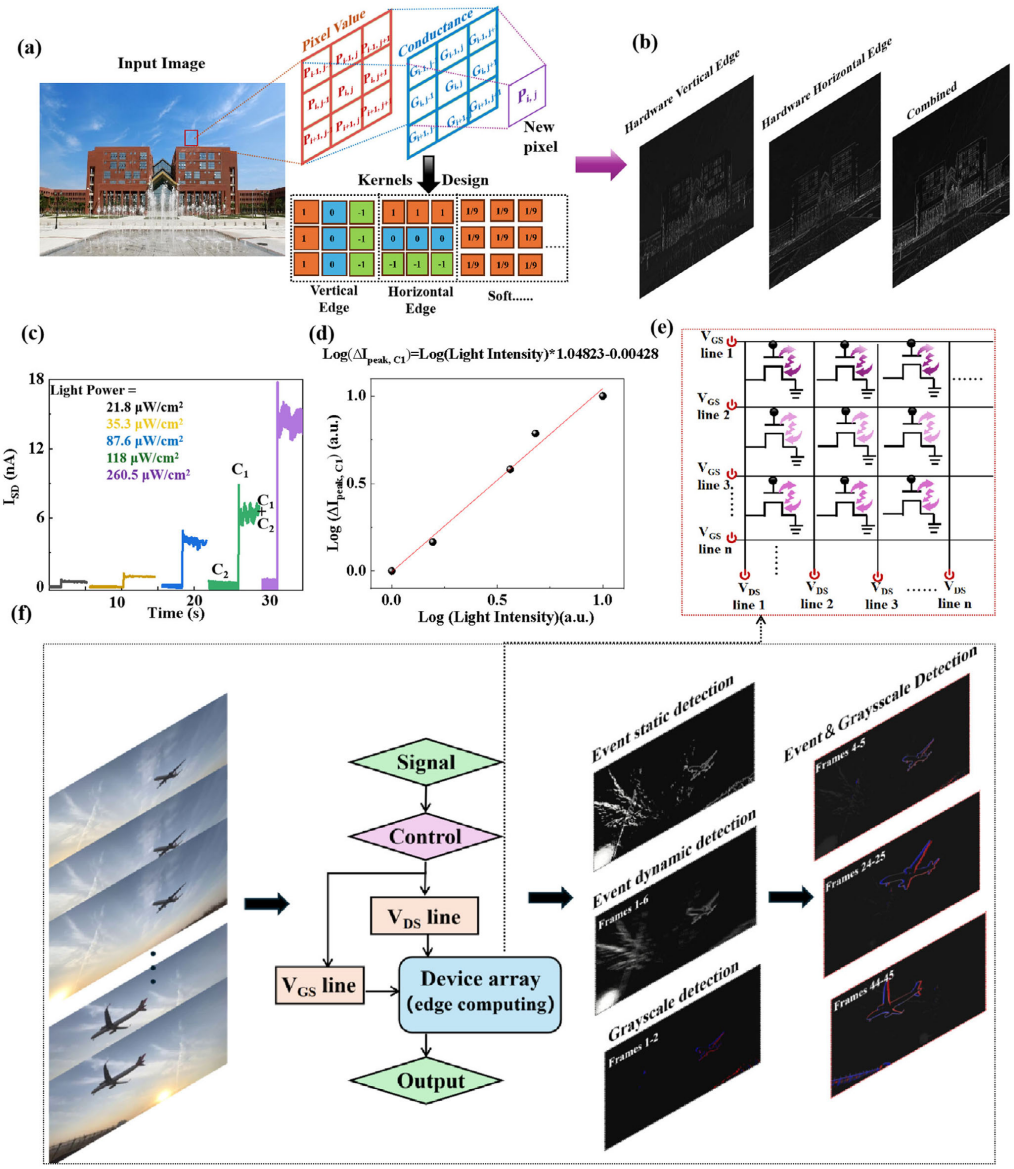
Applications of the piezo‐phototronic OAMT in image sensing and processing. (a) Details about hardware image processing procedure. The original input image of the school library situated in Tianjin University of Technology (TJUT). (b) The processed images using OAMT‐based hardware with different kernels, including vertical edge, horizontal edge and combined processes. (c) Adaptive photoresponse memory behavior of the device at different UV‐light power levels. (d) The calculated peak current values (C1–C2) were linearly fitted to the sensing light power density range, which corresponds to grayscale values. A grayscale threshold (light density) of 35.3 µW/cm^2^ was set, with points exceeding this threshold (the current equivalent to 35.3 µW/cm^2^) shown in red and those below it in blue. (e) The equivalent circuit diagram of the array. (f) Illustration of motion detection with the device array, including event and event's driven grayscale information.

Figure 7a exhibits the schematic diagram of hardware‐based convolutional image detection, [31] which a convolution operation is applied across the 3 × 3 conductance matrix programmed for each pixel in the original image. Multiply‐and‐accumulate (MAC) operations within the same column are performed uring the convolution operation by directly measuring the output conductance. The output conductance from each column is summed arithmetically, yielding the final output conductance (G_i,j_) at the corresponding pixel position (P_i,j_). The conductance maps are subsequently reorganized and visualized acting on the processed output image. The conductance matrix is derived from a 3 × 3 convolution matrix during actual kernel programming, and the sign of each matrix element is determined by the stable conductance difference between C2 (HRS) and C1+C2 (LRS) based on the LTP/LTD curves [32, 33]. During this stage, the kernel values are programmed based on the conductance difference between HRS and LRS of each OAMT in the 3 × 3 device array, considering the variation of device‐to‐device. Conductance of LRS (G^LRS^) minus conductance of HRS (G^HRS^) refers to ‘1’, and G^HRS^‐G^LRS^ refers to ‘−1’ in the convolution kernel matrix. The horizontal edge kernel uses the same data as the vertical edge, but the input vector is transposed. The significant difference in conductivity values enables the subsequent enhancement of image features during edge detection simulations. The detailed equivalent conductance matrix for the designed three kernels is shown in Figure S8, representing essential filtering functions commonly used in image processing. As shown in Figure S9, the soft filter produces a blurred image by averaging each pixel value with its surrounding pixels. The varied edge sensing results arise from the kernel directions, as finite difference computations in the local regions are performed along different orientations (Figure 7b). Figure S10 and Figure 7b illustrate the detailed processing, demonstrating simultaneous horizontal and vertical edge detection, which indicates that the OAMT‐based array is robust to device variation and capable of efficient convolutional image detection. Moreover, to demonstrate precise timing synchronization control between event and grayscale sensing in our devices, we implement a horizontal/vertical edge‐enhanced kernel combined with a Gaussian kernel design, enabling simultaneous dynamic and static detection of flight profiles (Figures S11 and S12). This design is further integrated with an optimized grayscale scheme to enhance dynamic image processing. Figure 7c illustrates the unique adaptive photoresponse memory behaviors of the device at different UV‐light power levels. Under UV illumination, C2 of 3,6‐DATT transitions to C1, generating transient current spikes for grayscale detection. With stress‐assistance biasing, C1 shifts to a C1+C2 state, producing stable current changes for event dynamic/static detection. Consequently, efficient detection of both events and event‐driven grayscale information within visual scenes can be achieved using a single transistor channel. The calculated peak current values (C_1_–C_2_) are linearly fitted to the sensing light power density range, which the grayscale values were corresponds to light power density. The grayscale threshold is set at 35.3 µW/cm^2^, where points with increased *∆I* exceeding the threshold (the current corresponding to 35.3 µW/cm^2^) are displayed in red, and points with decreased values falling below the negative threshold are displayed in blue (Figure 7d). As expected, while the reliable aircraft is in motion, the output image generated by the device array contains clear trajectory detection information that captures the aircraft's movement across one or more time points, including both static and dynamic flight path data as well as grayscale detection (Figure 7e,f). This ensures accurate data fusion and prevents timing discrepancies, indicating our designed OAMT's potential application in air defense security.

## 3 Conclusions

In conclusion, we report an advanced piezo‐phototronic modulated OAMTs that integrate DAVIS pixel functionality in a single‐transistor structure using a single‐crystal PZT piezoelectric medium with optimized stress distribution. Attributed to the efficient multi‐field (i.e., stress, electric fields, and UV light) coupling charge‐transport mechanism, the OAMT shows an ultrahigh *γ* as high as 0.87 under a *SS* ∼ 200 mV/decade, a quick programming/erasing rate of 2 s, a high ON/OFF ratio over 10^5^ and a long retention time more than 10^4^ s, respectively. Moreover, high linearity and excellent gain/attenuation symmetry in LTP/LTD synaptic functions are demonstrated in the device, enabling a recognition accuracy of over 90% with strong fault tolerance to noise ratios exceeding 30% in the ANN system simulations. At last, owing to the piezo‐phototronic‐driven multistage phase‐transition behaviors of the device array to generate stable and transient current changes, dynamic motion and static background information can be simultaneously captured, thereby simulating real‐time flight attitude detection and motion tracking through in‐memory sensing and computing. The result may open a new era in the development of artificial vision sensors.

## 4 Experimental Section/Method

### 4.1 Materials Characterization

UV–vis spectra were obtained on SHIMADZU UV‐3600 UV–vis–NIR. Optical microscopy images were captured using a Nikon ECLIPSE Ci‐POL equipped with a blue filter. Atomic force microscopy (AFM) in intelligent mode was conducted with a Bruker Dimension Icon. X‐ray diffraction (XRD) patterns were obtained in reflection mode at 45 kV and 200 mA using Cu Kα radiation with a RIGAKU SMARTLAB9KW diffractometer. Cross sections of Pt/PZT/LNO/Pt/TiO_2_/SiO_2_/Si devices were prepared and observed by FIB (Focused‐Ion‐Beam, FEI Helios Nanolab 460HP) and HRTEM (Talos F200 X). The ferroelectric properties were characterized using the Radiant Technologies Multiferroic Test System (Precision Multiferroic II).

### 4.2 Device Fabrication and Characterization of stress‐driven OAMTs

A platinized Si wafer (Pt(111)/TiO_2_/SiO_2_/Si) was used to deposit the films of 100 nm LNO buffer layer and 1.5 µm PZT film with Zr/Ti ratio of 52/48. The thin‐film OAMT was fabricated on a prepared PZT film‐based substrate with a bottom‐gate and top‐contact configuration by vacuum‐deposited patterned Au (30 nm) on a 25 nm 3,6‐DATT phase‐change semiconductor layers as source and drain electrodes. Semiconductor films of 25 nm pentacene and 3,6‐DATT were vacuum deposited at a 0.01 nm/s deposition rate and 23°C substrate, respectively. Au electrodes were vacuum deposited at a rateof 0.2 nm/s, by using a patterned shadow mask, For the ferroelectric properties of Pt/PZT/LNO/Pt/TiO_2_/SiO_2_/Si, magnetron sputtering method was used to deposit 100 nm top Pt electrodes as TE with Pt target (99.999%) in Ar atmosphere, DC was 80 W, and a mask of 300 µm diameter. For the Si‐based 3,6‐DATT devices, SiO_2_/Si wafers containing 300 nm‐thick SiO_2_ layers were beforehand cleaned and used the OTS surface modification by vapor deposition method in a vacuum chamber, as detailed in ref.^10^. The electrical properties of the devices were recorded using a Keithley 4200 SCS and a PDA FS380 semiconductor parameter analyzer at a micromanipulator 6150 probe station under ambient conditions inside a glove box.

Mathematical formulas used in the qualitative analysis of organic molecular materials.

Equations (1)–(3):

(1) $$C_{ele}\;\left(r\right)=A_{ele}\;\exp(-\frac{{\left(x-X_{ele}\right)}^{2}}{2\sigma_{ele,x}^{2}}-\frac{{\left(y-Y_{ele}\right)}^{2}}{2\sigma_{ele,y}^{2}}-\frac{{\left(z-Z_{ele}\right)}^{2}}{2\sigma_{ele,z}^{2}})$$

(2) $$C_{hole}\;\left(r\right)=A_{hole}\;\exp(-\frac{{\left(x-X_{hole}\right)}^{2}}{2\sigma_{hole,x}^{2}}-\frac{{\left(y-Y_{hole}\right)}^{2}}{2\sigma_{hole,y}^{2}}-\frac{{\left(z-Z_{hole}\right)}^{2}}{2\sigma_{hole,z}^{2}})$$

(3) $$\sigma_{hole,x}=\sqrt{\int {\left(x-X_{hole}\right)}^{2}\rho^{hole}(r)dr}\;$$

In the above equation, *A* is the normalized coefficient, such that both the *C_ele_* and *C_hole_* full‐space integral are 1. The variables *x*, *y*, and *z* represent the three Cartesian components of the coordinate vector *r*. *X*_ele_ refers to the X coordinate of the center of mass of the electrons. *σ* can be defined for both holes and electrons. The *x*, *y*, and *z* of *σ* are equivalent to the root‐mean‐square deviation (RMSD) of holes or electrons in the x, y, and z direction, reflecting the breadth of distribution or the degree of dispersion. The significance of defining *C*_hole_ and *C*_ele_ lay in the fact that the distribution of holes and electrons is often complex, which complicates graphical analysis. *C*_hole_ and *C*_ele_ represent the distribution of holes and electrons using a Gaussian function, thereby smoothing out distribution details. At this time, the equivalent map of holes and electrons becomes elliptical, which is obviously more clearly and intuitive during graphical investigation. If the *C*_hole_ and *C*_ele_ isoplane looks close to circular, the holes or electrons extend in a similar fashion in all directions. If the isoplane looks clearly elliptical, it distributes more along the long axis than along the other directions. Moreover, for the *C*_hole_ and *C*_ele_ isoplane maps, their positive center position is exactly the centroid position.

Equations (4) and (5):

(4) $$\mathrm{HDI}=100\times \sqrt{\int {\left[\rho^{\mathrm{hole}}\left(r\right)\right]}^{2}\mathrm{dr}}$$

(5) $$\mathrm{EDI}=100\times \sqrt{\int {\left[\rho^{\mathrm{ele}}\left(r\right)\right]}^{2}\mathrm{dr}}$$

The excited state wave function is integrated in full space to obtain *HDI* and *EDI*. The smaller the value, the higher the degree of hole/electron delocalization.

## Conflicts of Interest

The authors declare no conflicts of interest.

## Supporting information




**Supporting File**: advs74201‐sup‐0001‐SuppMat.docx.

## Data Availability

The data that support the findings of this study are available from the corresponding author upon reasonable request.
