# Supplementary material for: Single‐Crystal PZT‐Driven Organic Piezo‐Phototronic Adaptive Transistors Toward Advanced Spatiotemporal Visual Computing
Source: Adv Sci (Weinh). 2026 Feb 3;13(20):e21549. doi: 10.1002/advs.202521549 (PMC13067820; doi:10.1002/advs.202521549)
Supplement: Supplementary file 1 — Supporting File: advs74201‐sup‐0001‐SuppMat.docx. [file ADVS-13-e21549-s001.docx]

**Single-Crystal PZT-Driven Organic Piezo-Phototronic Adaptive Transistors toward Advanced Spatiotemporal Visual Computing**

*Chenhao Xu, Xingyu Cao, Zewen Li,* *Shifu Xiong*, Yongxu Hu***, Zhongwu Wang, Yujie Yuan*, Lei Zheng* and Wenping Hu*

C. Xu, Y. Yuan and L. Zheng

Tianjin Key Laboratory of Film Electronic and Communication Devices, School of Integrated Circuit Science and Engineering, Tianjin University of Technology, Tianjin 300384, China

X. Cao and S. Xiong

State Key Laboratory of Crystal Materials, Tianjin Key Laboratory of Functional Crystal Materials, Institute of Functional Crystals, Tianjin University of Technology Tianjin 300384, China

Z. Li

School of Materials Science and Engineering, Tianjin University of Technology, Tianjin 300384, China

Y. Hu, Z. Wang, and W. Hu,

Key Laboratory of Organic Integrated Circuits, Ministry of Education, Tianjin Key Laboratory of Molecular Optoelectronic Sciences, Department of Chemistry, Institute of Molecular Aggregation Science, Tianjin University, Tianjin 300072, China

^*^Corresponding e-mails: [xsf_optics@email.tjut.edu.cn;](mailto:xsf_optics@email.tjut.edu.cn;) [huyongxu@tju.edu.cn;](mailto:huyongxu@tju.edu.cn;) [yjyuan@email.tjut.edu.cn;](mailto:yjyuan@email.tjut.edu.cn;) [leizheng@tju.edu.cn;](mailto:wangzw@tju.edu.cn;)


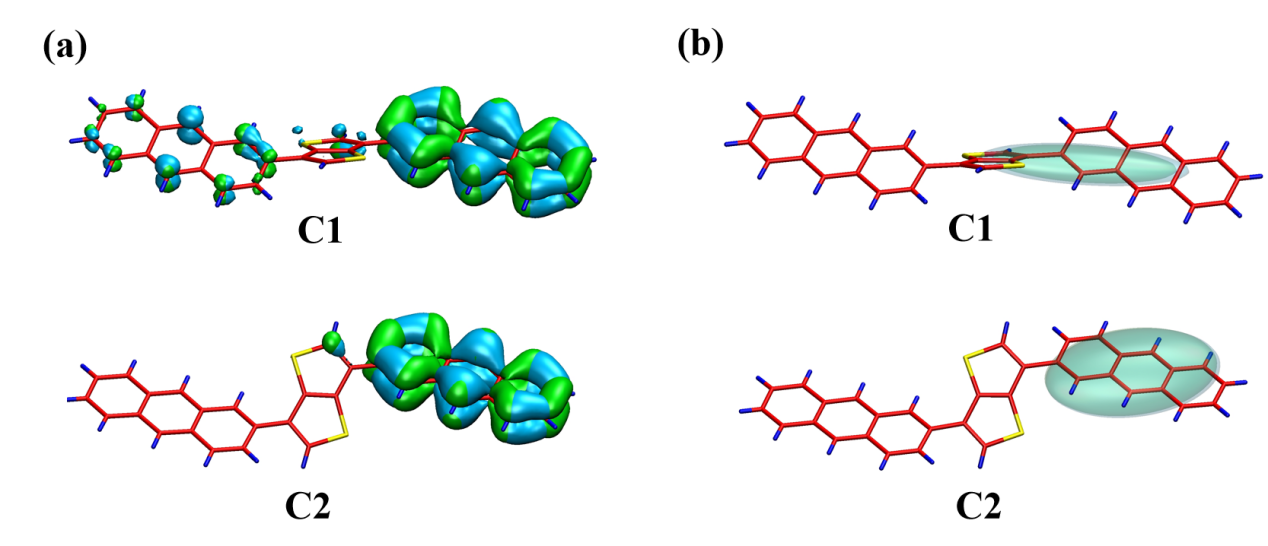


**Figure S1.** (a) Electron-hole distribution isosurface, holes are enriched in blue partial and electrons are enriched in green partial of C1 and C2. (b) The distribution of holes and electrons of C1 and C2 equally with a Gaussian function.


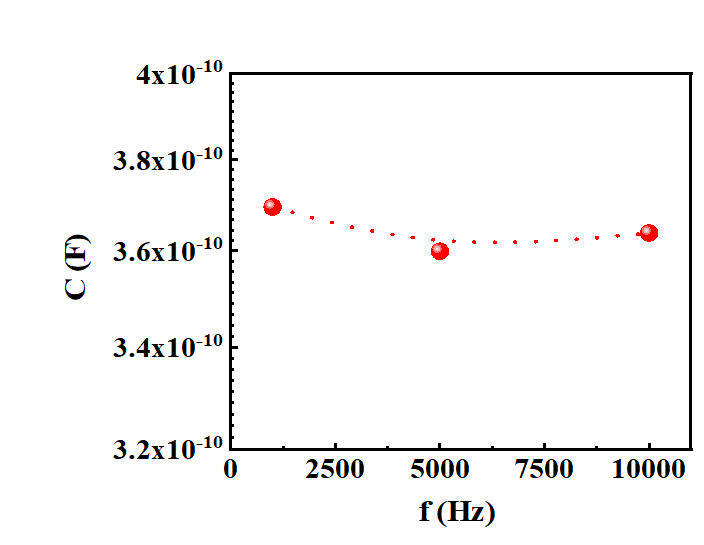


**Figure S2.** Capacitance-frequency (C-f) measured at different frequencies of the PZT film with 1.5μm thickness. ( bias voltages = ±1V).


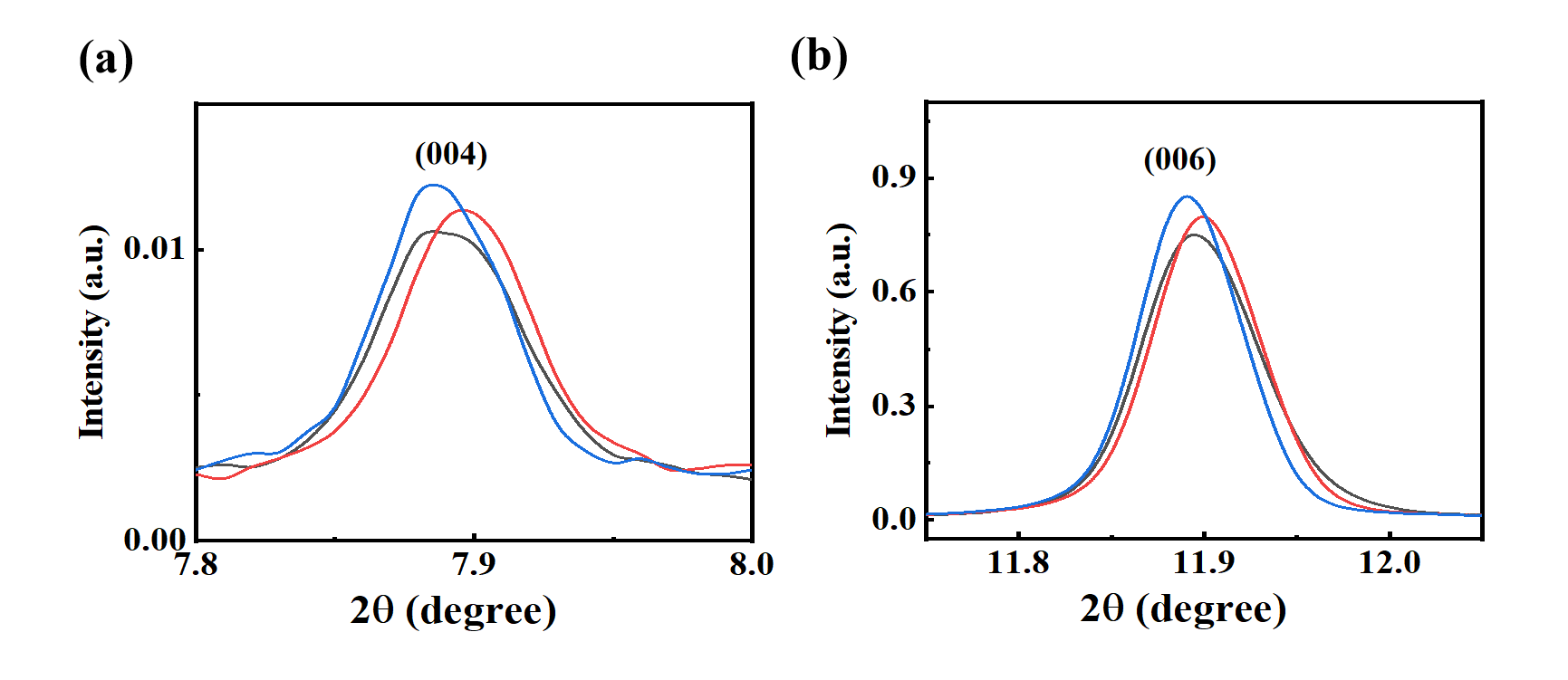


**Figure S3.** In-plane XRD patterns of 3,6-DATT film before and after stress/UV stimuli at the large *θ*.


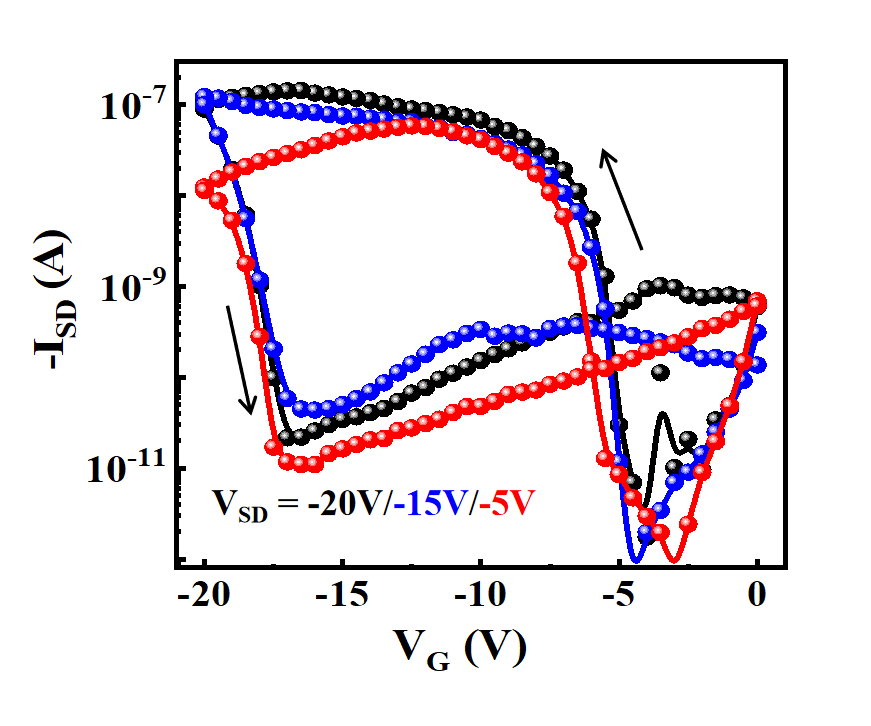


**Figure S4.** The hysteresis loops measured at different *V_SD_* conditions.

**Table S1.** Comparison of the Memory Characteristics of Representative Organic Memories in Recent Years.

| Memory mechanism | Active materials | Capacity factor  (γ) | Subthreshold  swing  (*SS,* V/decade) | I_ON_/I_OFF_ | Retention times (s) | Ref |
| --- | --- | --- | --- | --- | --- | --- |
| free-trapping layer (Single organic semiconductor) | BBTNDT | 0.38 | No | 10^6^ | >10^4^ | [1] |
|  | 2,7-DAN | 0.33 | No | 10^9^ | >10^4^ | [2] |
|  | Pentacene | 0.27 | No | 10^4^ | >10^4^ | [3] |
|  | 3,6-DATT | 0.38 | No | 10^7^ | >10^4^ | [4] |
| free-trapping layer (Multilayer organic semiconductor) | Pentacene/  MAPbBr_3_ | 0.08 | No | > 10^4^ | >10^4^ | [5] |
|  | CuPc/p-6P | 0.53 | No | > 10^4^ | No | [6] |
|  | Pentacene/P13/  Pentacene | 0.6 | No | 10^4^ | >10^4^ | [7] |
|  | IDTBT:N2200 | 0.72 | No | 10^5^ | >10^4^ | [8] |
|  | PTDPPTFT4:o-MeO  -DMBI on Si | 0.12 | No | 10^4^ | >10^5^ | [9] |
|  | PTDPPTFT4:o-MeO-DMBI on BCB/Si | 0.11 | No | 10^4^ | >10^5^ | [9] |
|  | PTDPPTFT4:o-MeO-DMBI OTS/Si | 0.24 | No | 10^4^ | >10^5^ | [9] |
| Electret/ferroelectric  dielectric | DNTT/PS | 0.47 | 1.5 | > 10^5^ | >10^4^ | [10] |
|  | PTCDI-C13/PI | 0.61 | No | 10^4^ | 10^2^ | [11] |
|  | TIPS-pentacene/  syn-B2IPIO/PS | 0.65 | No | 10^5^ | 10^4^ | [12] |
|  | DPP-DTT/PVN | 0.55 | No | 10^3^ | 10^4^ | [13] |
|  | C8-BTBT/  P(DF-TrFE) | 0.39 | No | >10^3^ | 10^4^ | [14] |
|  | Pentacene  /P(DF-TrAFE) | 0.56 | No | 10^3^ | **>**10^4^ | [15] |
|  | TIPS-pentacene/  P(DF-TrAFE) | 0.4 | 1.36 | 10^3^ | **>**10^4^ | [16] |
| Floating gate | HfO_2_/CuPc/N-C_60_  /PVP/Pentacene | 0.44 | No | 10^4^ | 10^4^ | [17] |
|  | SiO_2_/GO/PMMA  /PbSe | 0.12 | No | 10 | 10^4^ | [18] |
|  | Al_2_O_3_/PVP:QDs/  Al_2_O_3_/Ag nanowires/PDVT-8 | 0.85 | No | 10^4^ | 10^8^ | [19] |
|  | P(DF-TrAFE-CFE)/FBBT:PS/C_10_-DNTT | 0.25 | No | 10^5^ | **>**10^4^ | [20] |
| **Stress driven** | **3,6-DATT** | **0.87** | **0.2** | **> 10^5^** | **>10^4^** | **This work** |

Notes: The *γ* is the corresponding ratio of memory window/working voltage.


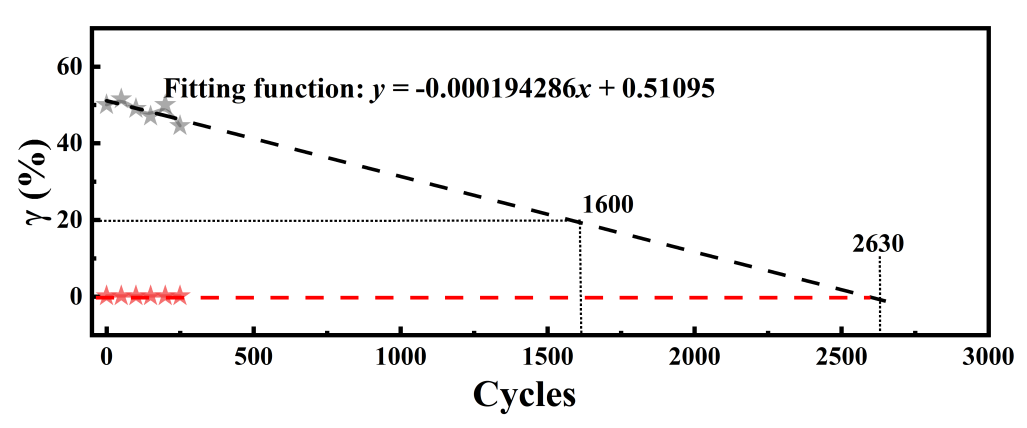


**Figure S5.** Cyclability testing and fitting analysis.


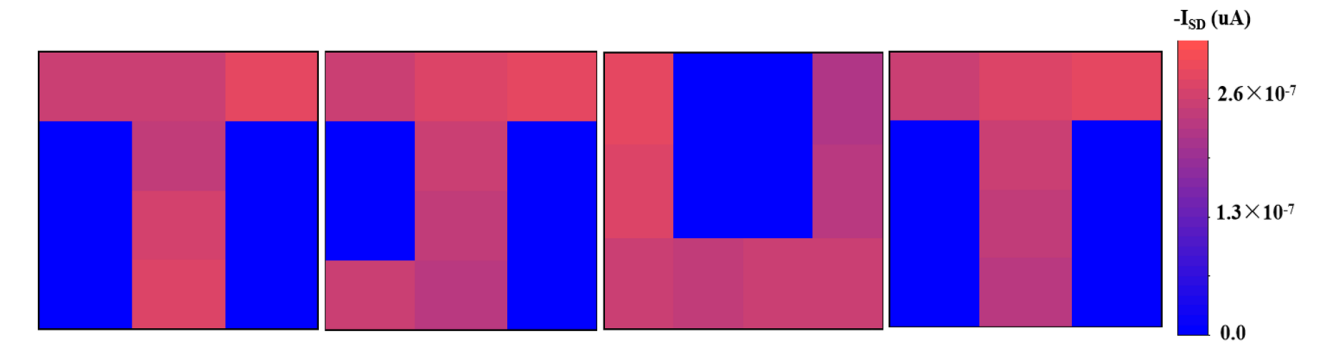


**Figure S6.** The stability of the device continuous operation reach up to 1 hours with a slightly decreased currents.


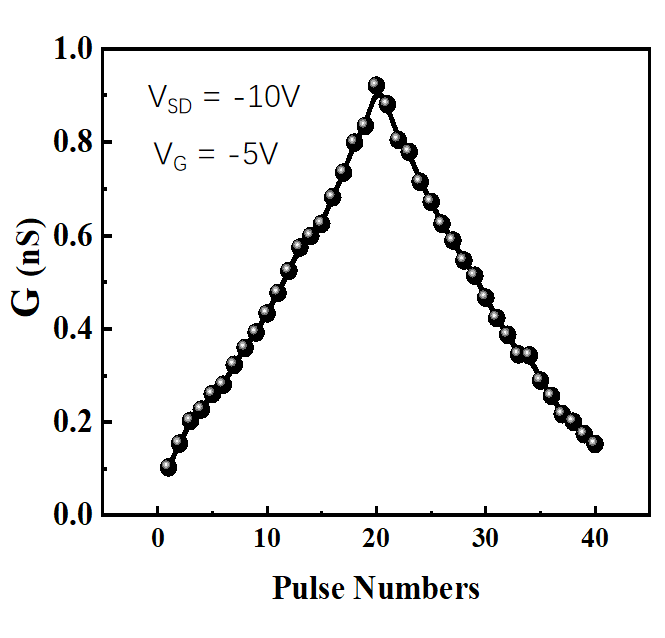


**Figure S7.** LTP/LTD behavior using stress-effect OPCMT.


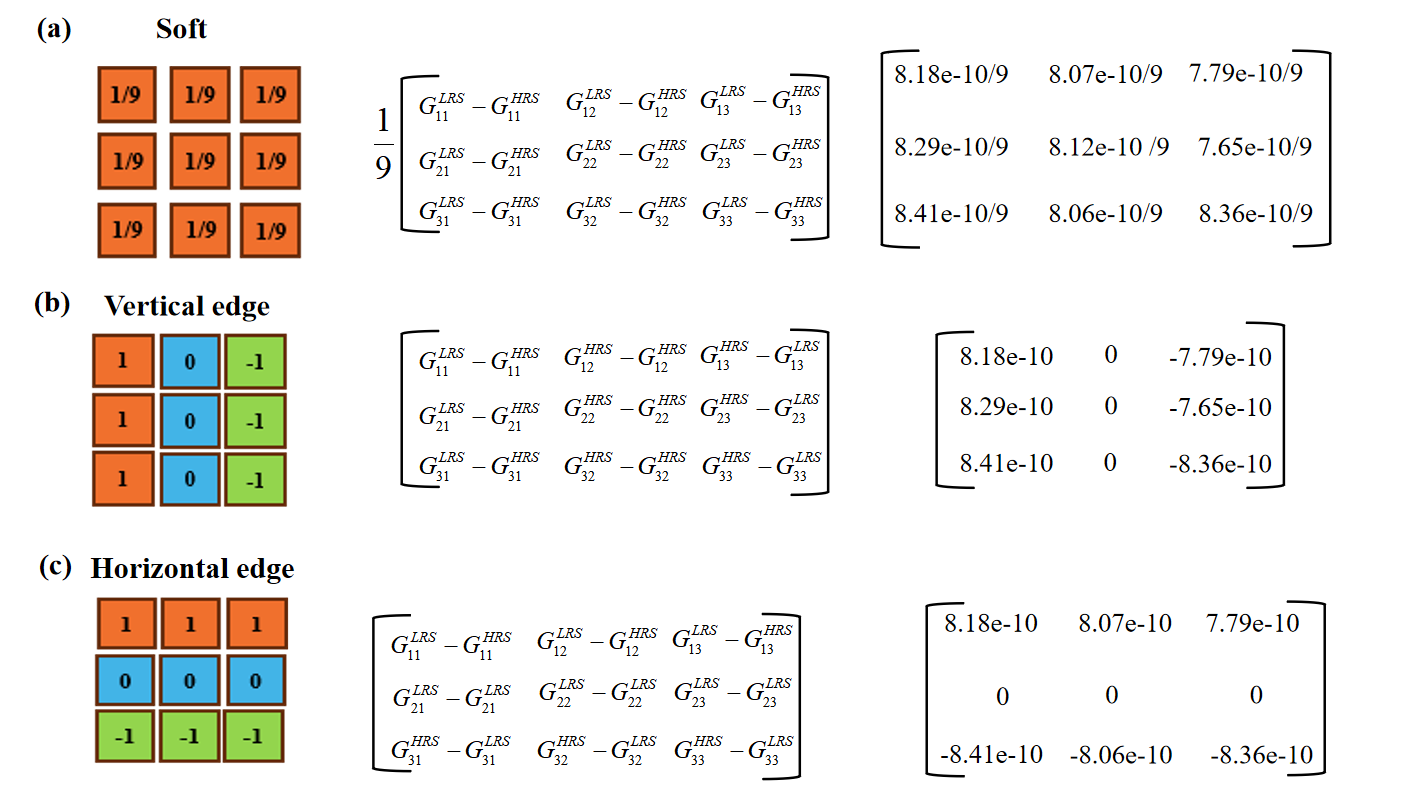


**Figure S8.** (a-c) Kernel design of image processing, including soft, horizontal and vertical edges. The ideal convolution kernel matrix and the corresponding equivalent conductance matrix. Conductance of LRS (G_LRS_) minus conductance of HRS (G_HRS_) refers to‘1’, and G_HRS_-G_LRS_ refers to ‘-1’ in the convolution kernel matrix. The horizontal edge kernel uses the same data as the vertical edge, but the input vector is transposed.


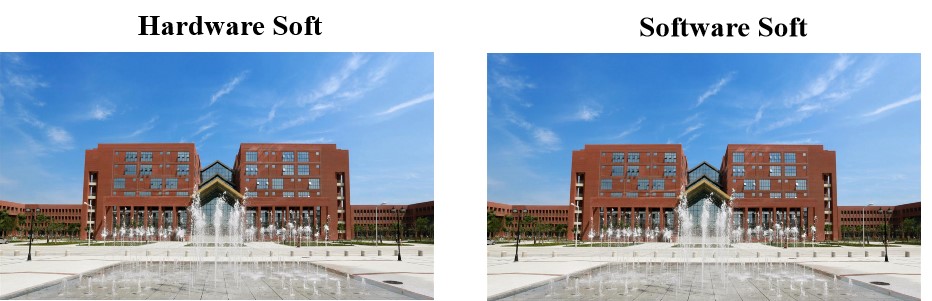


**Figure S9.** The processed images using OPCMT-based hardware with soft kernel.


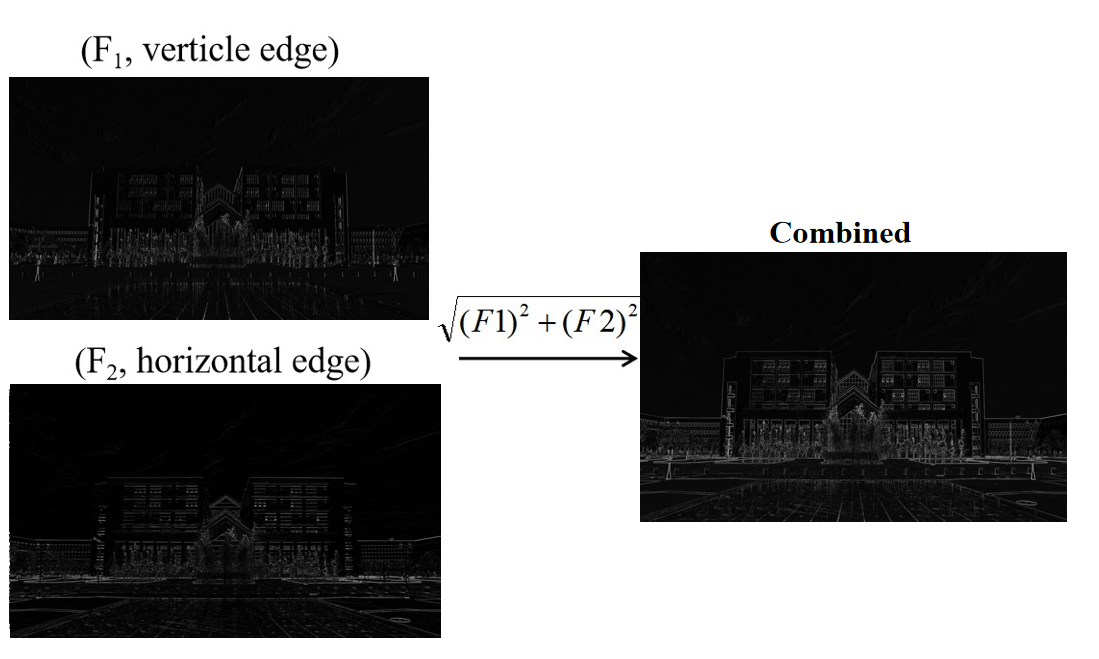


**Figure S10.** Schematic of edge combined figure in Figure 6g and 6j.


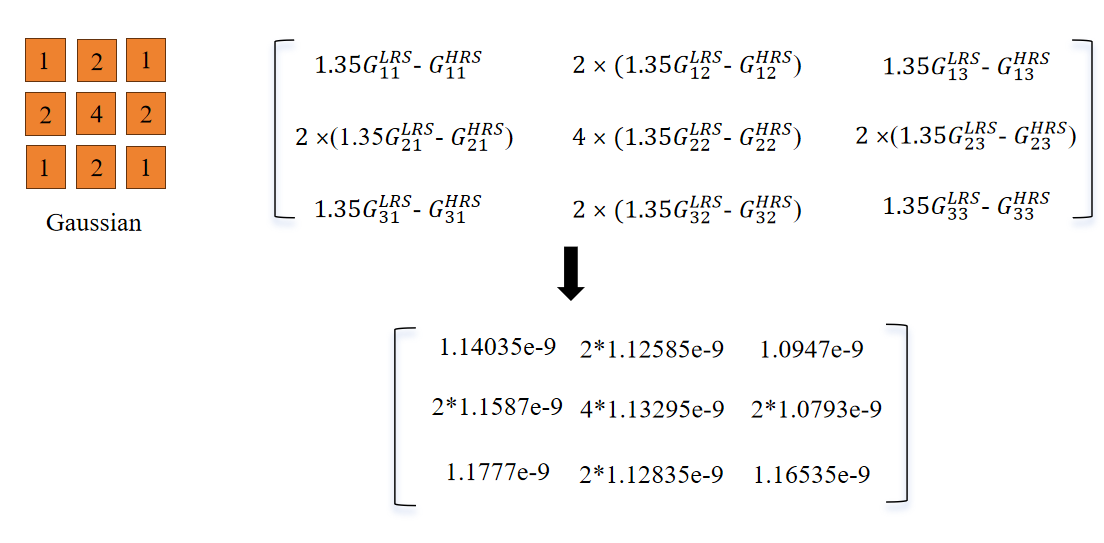


**Figure S11.** Gaussian kernel design of image processing and their ideal convolution kernel matrix and the corresponding equivalent conductance matrix.


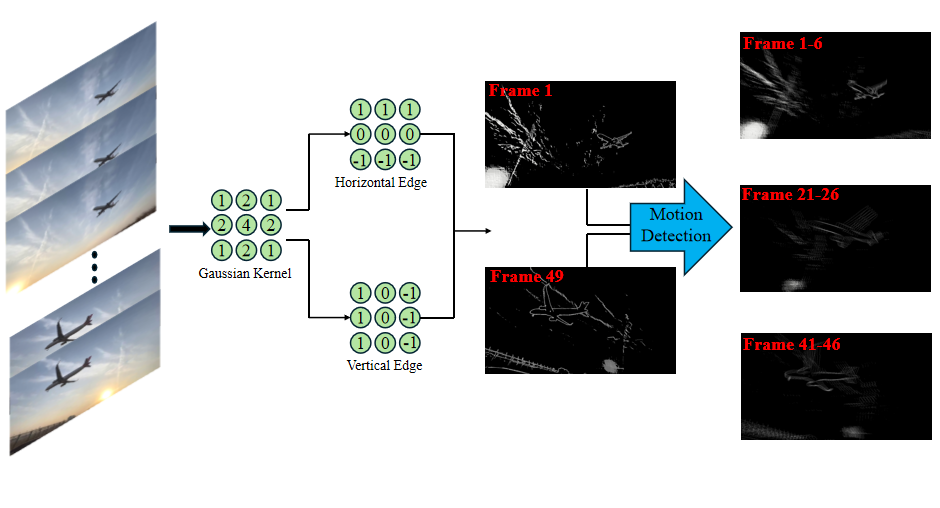


**Figure S12.** Illustration of motion detection including event dynamic and static information.

**References**

1. K. Pei, X. Ren, Z. Zhou, Z. Zhang, X. Ji, and P. K. L. Chan, “A High-Performance Optical Memory Array Based on Inhomogeneity of Organic Semiconductors,” *Advanced Materials* 30 (2018): 1706647, [https://doi.org/10.1002/adma.201706647.](https://doi.org/10.1002/adma.201706647)
2. L. Zheng, J. Li, Y. Wang, et al., “High-Performance Optical Memory Transistors Based on a Novel Organic Semiconductor with Nanosprouts,” *Nanoscale* 11 (2019): 7117-7122, [https://doi.org/10.1039/C9NR00578A.](https://doi.org/10.1039/C9NR00578A)
3. Y. Hu, L. Zheng, J. Li, et al., “Organic Phase-Change Memory Transistor Based on an Organic Semiconductor with Reversible Molecular Conformation Transition,” *Advanced Science* 10 (2023): 2205694, [https://doi.org/10.1002/advs.202205694.](https://doi.org/10.1002/advs.202205694)
4. X. Liu, H. Zhao, G. Dong, et al., “Multifunctional Organic Phototransistor-Based Nonvolatile Memory Achieved by UV/Ozone Treatment of the Ta_2_O_5_ Gate Dielectric,” *ACS Applied Materials & Interfaces* 6 (2014): 8337-8344, [https://doi.org/10.1021/am501197d.](https://doi.org/10.1021/am501197d)
5. J. Chen, Y. Chiu, Y. Li, C. Chueh, and W. Chen, “Nonvolatile Perovskite-Based Photomemory with a Multilevel Memory Behavior,” *Advanced Materials* 29 (2017): 1702217, [https://doi.org/10.1002/adma.201702217.](https://doi.org/10.1002/adma.201702217)
6. C. Qian, J. Sun, L. A. Kong, et al., “High-Performance Organic Heterojunction Phototransistors Based on Highly Ordered Copper Phthalocyanine/Para-Sexiphenyl Thin Films,” *Advanced Functional Materials* 27 (2017): 1604933, <https://doi.org/10.1002/adfm.201604933>.
7. W. Li, F. Guo, H. Ling, et al., “High-Performance Nonvolatile Organic Field-Effect Transistor Memory Based on Organic Semiconductor Heterostructures of Pentacene/P13/Pentacene as Both Charge Transport and Trapping Layers,” *Advanced Science* 4 (2017): 1700007, <https://doi.org/10.1002/advs.201700007>.
8. S. Lan, J. Zhong, E. Li, et al., “High-Performance Nonvolatile Organic Photoelectronic Transistor Memory Based on Bulk Heterojunction Structure,” *ACS Applied Materials & Interfaces* 12 (2020): 31716-31724, <https://doi.org/10.1021/acsami.0c09221>.
9. W. Y. Lee, H. C. Wu, C. Lu, B. D. Naab, W. C. Chen, and Z. Bao, “n-Type Doped Conjugated Polymer for Nonvolatile Memory,” *Advanced Materials* 29 (2017): 1605166, <https://doi.org/10.1002/adma.201605166>.
10. X. Ren, and P. K. L. Chan, “23 Bits Optical Sensor Based on Nonvolatile Organic Memory Transistor,” *Applied Physics Letters* 104 (2014), <https://doi.org/10.1063/1.4869308>.
11. W. Chou, S. Peng, F. Wu, et al., “Memory Characteristics of Organic Field-Effect Memory Transistors Modulated by Nano-p–n Junctions,” *Journal of Materials Chemistry C* 8 (2020): 7501-7508, <https://doi.org/10.1039/D0TC01233E>.
12. D. Liu, Y. Zhang, X. Li, et al., “Nonvolatile Organic Field-Effect Transistor Memory from Pyrene-Fused Azaindacene Regioisomers,” *Journal of Materials Chemistry C* 9 (2021): 6560-6567, <https://doi.org/10.1039/D1TC00560J>.
13. X. She, D. Gustafsson, and H. Sirringhaus, “A Vertical Organic Transistor Architecture for Fast Nonvolatile Memory,” *Advanced Materials* 29 (2017): 1604769, <https://doi.org/10.1002/adma.201604769>.
14. M. Xu, C. Zhao, Z. Meng, et al., “Nonvolatile Memory Organic Light-Emitting Transistors,” *Advanced Materials* 35 (2023): 2307703, <https://doi.org/10.1002/adma.202307703>.
15. M. Kang, S. A. Lee, S. Jang, et al., “Low-Voltage Organic Transistor Memory Fiber with a Nanograined Organic Ferroelectric Film,” *ACS Applied Materials & Interfaces* 11 (2019): 22575-22582, <https://doi.org/10.1021/acsami.9b03564>.
16. S. Rahi, V. Raghuwanshi, G. Konwar, and S. P. Tiwari, “High-Performance Flexible Solution-Processed Organic Nonvolatile Memory Transistors,” *IEEE Transactions on Electron Devices* 70 (2023): 4338-4344, <https://doi.org/10.1109/TED.2023.3283346>.
17. H. Chang, C. Lu, C. Liu, and W. Chen, “Single-Crystal C_60_ Needle/CuPc Nanoparticle Double Floating-Gate for Low-Voltage Organic Transistors Based Non-Volatile Memory Devices,” *Advanced Materials* 27 (2015): 27-33, <https://doi.org/10.1002/adma.201403771>.
18. Y. Che, Y. Zhang, X. Cao, et al., “Low Operating Voltage Ambipolar Graphene Oxide-Floating-Gate Memory Devices Based on Quantum Dots,” *Journal of Materials Chemistry C* 4 (2016): 1420-1424, <https://doi.org/10.1039/C5TC04007H>.
19. D. Hu, X. Wang, H. Chen, and T. Guo, “High Performance Flexible Nonvolatile Memory Based on Vertical Organic Thin Film Transistor,” *Advanced Functional Materials* 27 (2017): 1703541, <https://doi.org/10.1002/adfm.201703541>.
20. T. Xu, S. Guo, W. Qi, S. Li, M. Xu, and W. Wang, “Organic Transistor Nonvolatile Memory with Three-Level Information Storage and Optical Detection Functions,” *ACS Applied Materials & Interfaces* 12 (2020): 21952-21960, <https://doi.org/10.1021/acsami.0c01162>.
